# Discrete finger sequences are widely represented in human striatum

**DOI:** 10.1038/s41598-020-69923-x

**Published:** 2020-08-06

**Authors:** Kasper Winther Andersen, Kristoffer H. Madsen, Hartwig Roman Siebner

**Affiliations:** 1 Danish Research Centre for Magnetic Resonance (DRCMR), Centre for Functional and Diagnostic Imaging and Research, Copenhagen University Hospital Hvidovre, Kettegård Allé 30, 2650 Hvidovre, Denmark; 2 Department of Applied Mathematics and Computer Science, Technical University of Denmark, Kgs. Lyngby, Denmark; 3 Institute for Clinical Medicine, Faculty of Medical and Health Sciences, University of Copenhagen, Copenhagen, Denmark; 4 Department of Neurology, Copenhagen University Hospital Bispebjerg, Copenhagen, Denmark

**Keywords:** Motor control, Basal ganglia

## Abstract

Research in primates and rodents ascribes the striatum a critical role in integrating elementary movements into unitary action sequences through reinforcement-based learning. Yet it remains to be shown whether the human striatum represents action sequence-specific information. Young right-handed volunteers underwent functional magnetic resonance imaging while they performed four discrete finger sequences with their right hand, consisting of five button presses. Specific finger sequences could be discriminated based on the distributed activity patterns in left and right striatum, but not by average differences in single-voxel activity. Multiple bilateral clusters in putamen and caudate nucleus belonging to motor, associative, parietal and limbic territories contributed to classification sensitivity. The results show that individual finger movement sequences are widely represented in human striatum, supporting functional integration rather than segregation. The findings are compatible with the idea that the basal ganglia simultaneously integrate motor, associative and limbic aspects in the control of complex overlearned behaviour.

## Introduction

Humans have an extraordinary capacity of acquiring novel manual skills throughout life and improve their accuracy, speed, and efficiency through repeated practice. Manual skills such as handwriting or tying a shoe are characterized by highly overlearned sequences of elementary actions, which can be produced with a high degree of automaticity. The question how humans learn novel motor skills has been addressed in numerous functional neuroimaging studies in which healthy volunteers learned novel sequences of discrete finger movements. These studies identified a set of brain regions, including frontoparietal cortical areas, putamen and cerebellum involved in motor sequence learning^1,2^. In the putamen and frontoparietal areas, task-related activity shifts from associative to sensorimotor territories, as performance becomes more and more automatic^3–6^. This gradual shift in activation shows that motor sequence learning initially engages visuospatial allocentric representations and relies more and more on egocentric sensorimotor representation at later learning stages. Other neuroimaging studies have implicated the sensorimotor putamen in memory consolidation of motor sequences^7,8^ and chunking of elemental actions^9^. Taken together, neuroimaging data point to a pivotal role of the striatum in learning and executing motor sequences.

Invasive studies in monkeys confirm a critical contribution of the basal ganglia^10,11^. A reversible disruption of striatal activity by local injections of the GABA agonist muscimol impaired the learning of new sequences when targeting the anterior caudate nucleus and putamen^12^. The execution of well-learned sequences was disrupted after injections in the middle-posterior putamen and, less severely, after injections in the anterior caudate nucleus and putamen^12^. In rodents, marked changes in spike activity patterns occur in the associative and sensorimotor striatum with a preferential engagement of the associative territory early in training and the sensorimotor territory later in training^13^. When learning rapid action sequences, striatal projection neurons and interneurons express specific sequence-related activity^14,15^. Projection neurons may preferentially fire at sequence initiation and termination or may display sustained or suppressed spiking activity levels throughout the learned action sequence, suggesting that sequences are represented as a single chunk in the striatum^14^. Further, the corticostriatal dynamics of cell firing reflect the refinement of behavioural features that were reinforced during learning^16^. While striatal projection neurons mark the sequence-boundary, fast-spiking interneurons show an inverse pattern, firing between the initiation and termination of the learned motor sequences^15^.

In recent years, multivariate pattern analysis (MVPA) has emerged as a powerful method to infer sensory, motor and cognitive representations from distributed brain activity patterns as revealed by functional MRI^17–19^. A seminal paper showed that sequence-specific activation patterns were strengthened by learning, becoming more distinct relative to non-learned sequences^20^. More recent studies suggest that premotor areas rather than primary motor cortex appear to represent motor sequences during preparation and execution^21–23^. Despite of the importance of the basal ganglia for skilled sequential movements, sequential movements could not be decoded from distributed activity in the basal ganglia^21, 24^ or analyses exclusively focused on the cortex^20, 22^. Yet, a recent study compared the distributed activity patterns associated between two new and two trained sequences^25^. In that study, not only prefrontal and secondary motor cortical areas but also the dorsolateral striatum showed greater representations for consolidated sequences than untrained ones. In addition, activity patterns differentiated the two new and learned sequences, pointing to a representation of motor sequences at both, the cortical and striatal level^25^.

## Results

**Figure 1:**
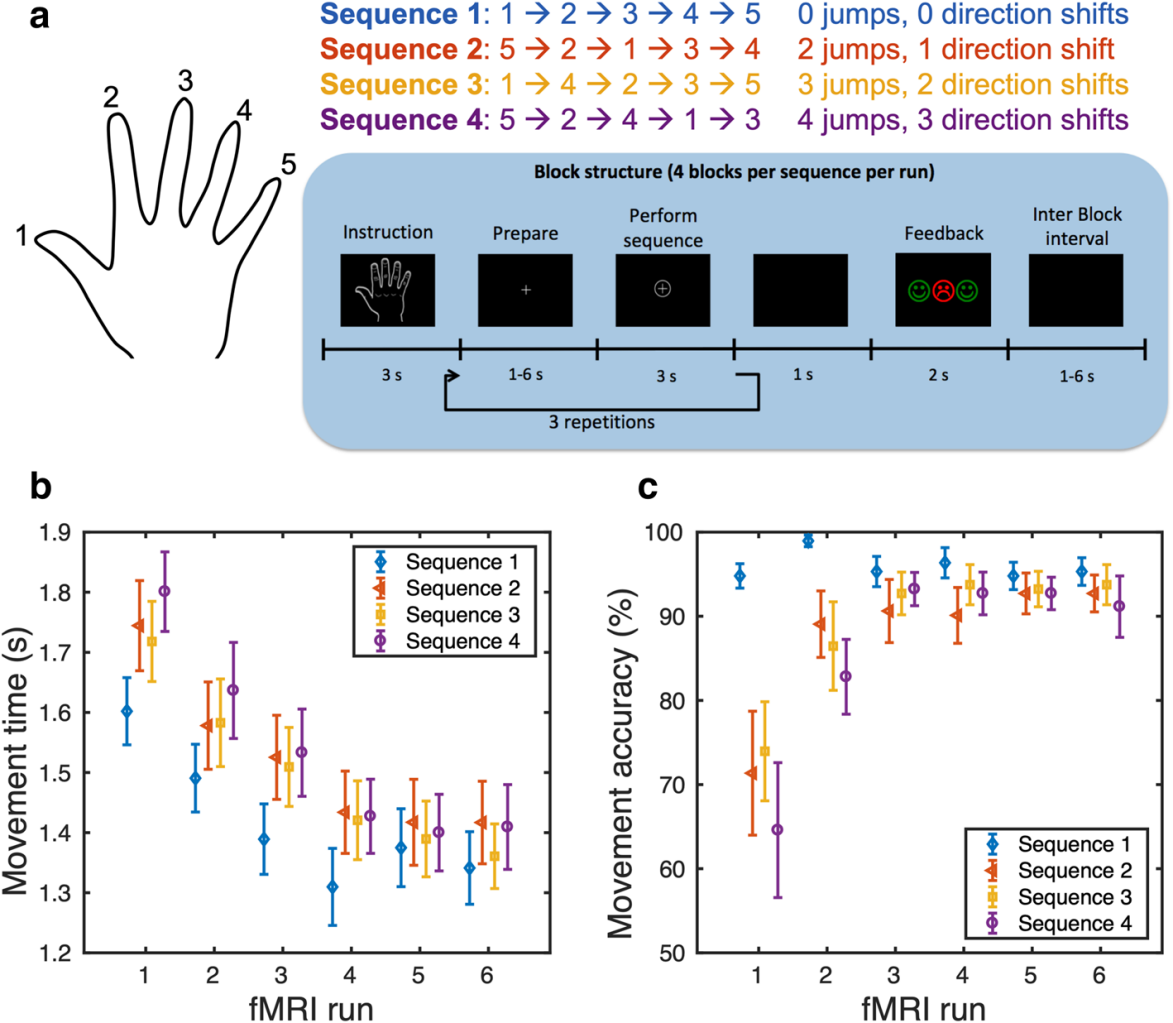
Behavioral results. **(a)** shows the 4 different sequences, which the subjects learned and performed during 6 fMRI runs. Mean (standard error bars) across n = 16 subjects for movement time (MT) **(b)** and movement accuracy **(c)** per run for each of the 4 different tapping-sequences. Stable speed and accuracy were found in the last three runs.

This study was designed to delineate how widespread motor sequences are represented in the human striatum. To this end, healthy young volunteers underwent six consecutive fMRI runs at 3 T while they learned and performed four 5-element finger sequences (Fig. 1a). Data analysis was restricted to those fMRI runs, during which performance had reached a plateau, indicating that subjects had become skilled in executing the sequences. While univariate analysis revealed no differences in voxel-wise activity levels between the learned sequences, multivariate pattern analysis revealed a widespread and sparse representation of the learned sequences in ipsi-lateral and contra-lateral striatum with no evidence for topographic clustering in the motor territory of the striatum.

### Behavioural results

Participants became gradually faster and more accurate to perform the finger sequences, see Fig. 1b,c. For movement time, this is reflected by a main effect of fMRI run (F(3, 97) = 63.3, p < 0.001). Mean total movement time monotonically decrease from run 1 to run 4 (pair-wise t-tests: p < 0.003), reaching a plateau for run 4 to 6 (pair-wise t-tests: p > 0.41). We also found a main effect of sequence (F(2.01,96.92) = 6.63, p = 0.004). Subjects were significantly faster at executing the regular sequence 1 relative to the more complex sequences 2–4 (pair-wise t-tests: p < 0.011). There were no differences in mean movement time among sequences 2, 3 and 4 (pair-wise t-tests: all p > 0.17). All sequences were also performed more accurately over the course of the experiment. For the movement accuracy, the ANOVA revealed a main effect of fMRI run (F(1.7, 89.1) = 8.2, p < 0.001), but also a main effect of motor sequence (F(2.5, 89.1) = 7.5, p = 0.001), and a run x sequence interaction (F(5.9, 89.1) = 3.0, p = 0.010). Post-hoc tests showed that performance in run 1 was significantly less accurate than performance in runs 2–6 (all p < 0.007). The fMRI runs 2–6 did not differ in terms of accuracy (all p > 0.152). Subjects were significantly more accurate when executing sequence 1 than sequences 2–4 (all p < 0.009), while there were no significantly differences between sequences 2–4 (all p > 0.202). The interaction between “run” and “sequence” was driven by subjects being significantly more accurate when performing sequence 1 compared with the other sequences in fMRI runs 1 and 2 (all p < 0.05). In the following we focus our analysis of the fMRI data on the last three runs (4–6) during which participants showed stable performance in terms of speed and accuracy.

### Multivariate striatal activity patterns code for sequences

The multivariate pattern of execution-related activity in both left and right striatum was significantly different between sequences. In left putamen, contralateral to movement, the classification accuracy was 40.6% (p_permutation_ < 0.001, chance level 25%), showing that the activity pattern can differentiate the different sequential movements. In left caudate nucleus the classification accuracy was 35.9% (p_permutation_ = 0.002). Considering execution-related activity patterns from left putamen and caudate nucleus together slightly increased the accuracy to 42.7% (p_permutation_ < 0.001), compared with left putamen alone. In right putamen and caudate nucleus, ipsilateral to movement, accuracies were 36.5% (p_permutation_ = 0.002) and 43.2% (p_permutation_ < 0.001), respectively. Combining right putamen and caudate nucleus (43.2%, p_permutation_ < 0.001) did not increase the accuracy compared with right caudate nucleus alone. All results survived false discovery rate (FDR) correction for multiple comparisons (q < 0.05). Sequence discriminations were not driven by the inclusion of the simple ascending or descending finger sequence (sequence 1: 1-2-3-4-5 or 5-4-3-2-1). When excluding this sequence from the analyses, classification accuracies between sequences 2–4 were still significant (left putamen 49.3% (p_permutation_ < 0.001), left caudate nucleus 45.1% (p_permutation_ = 0.003), left putamen and caudate nucleus 52.8% (p_permutation_ < 0.001), right putamen 41.7% (p_permutation_ = 0.024), right caudate nucleus 57.0% (p_permutation_ < 0.001), right putamen and caudate nucleus 52.8% (p_permutation_ < 0.001), chance level: 33%).

### Finger sequences are represented in small patches in human striatum

**Figure 2:**
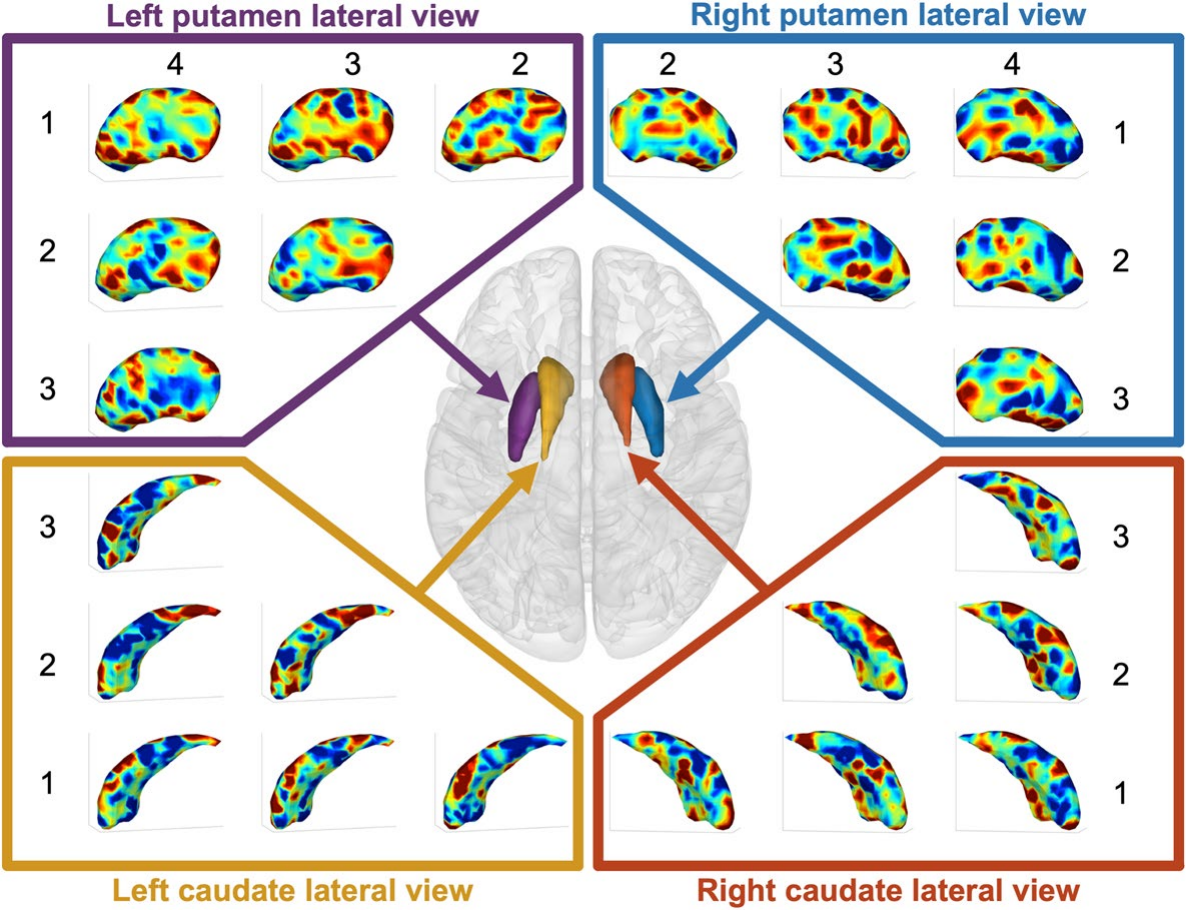
Classifier weight maps. Classifier weight maps for left and right putamen and caudate nucleus separately. The weight maps are constructed using one-against-one linear SVM classifiers between each pair of the 4 tapping-sequences. High positive (red) or negative (blue) weight are areas important for classification. The numbers indicate the sequence for which the classifiers where constructed, e.g. the top-left panel shows the weight map for the classifier discriminating between sequences 1 and 4. N = 192 samples used for classification. Figures were made using SPM12 software (https://www.fil.ion.ucl.ac.uk/spm/software/spm12).

**Figure 3:**
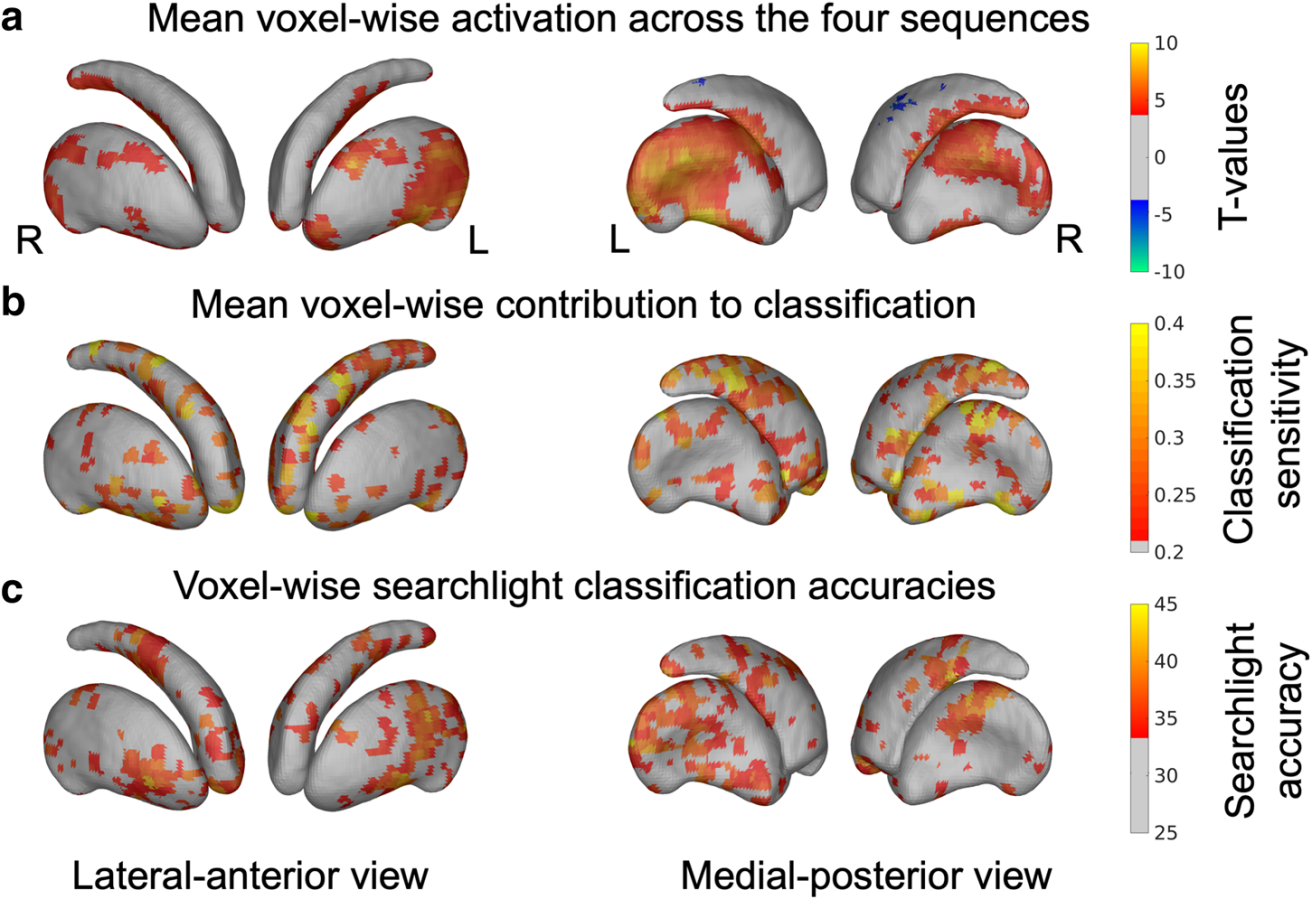
**(a-c)** Surface rendered statistical maps of the striatum. **(a)** Mean activation across the four different sequences rendered on the surface of putamen and caudate (n = 16 subjects). **(b)** Mean voxel sensitivity across classifiers using whole-structure classification. High values indicate areas important for classifying between tapping-sequences (n = 192 samples used for classification). **(c)** Searchlight classification accuracies when classifying between tapping-sequences using a sphere with 5 mm radius (n = 192 samples used for classification). L = Left; R = Right. Figures were made using SPM12 software (https://www.fil.ion.ucl.ac.uk/spm/software/spm12).

**Figure 4:**
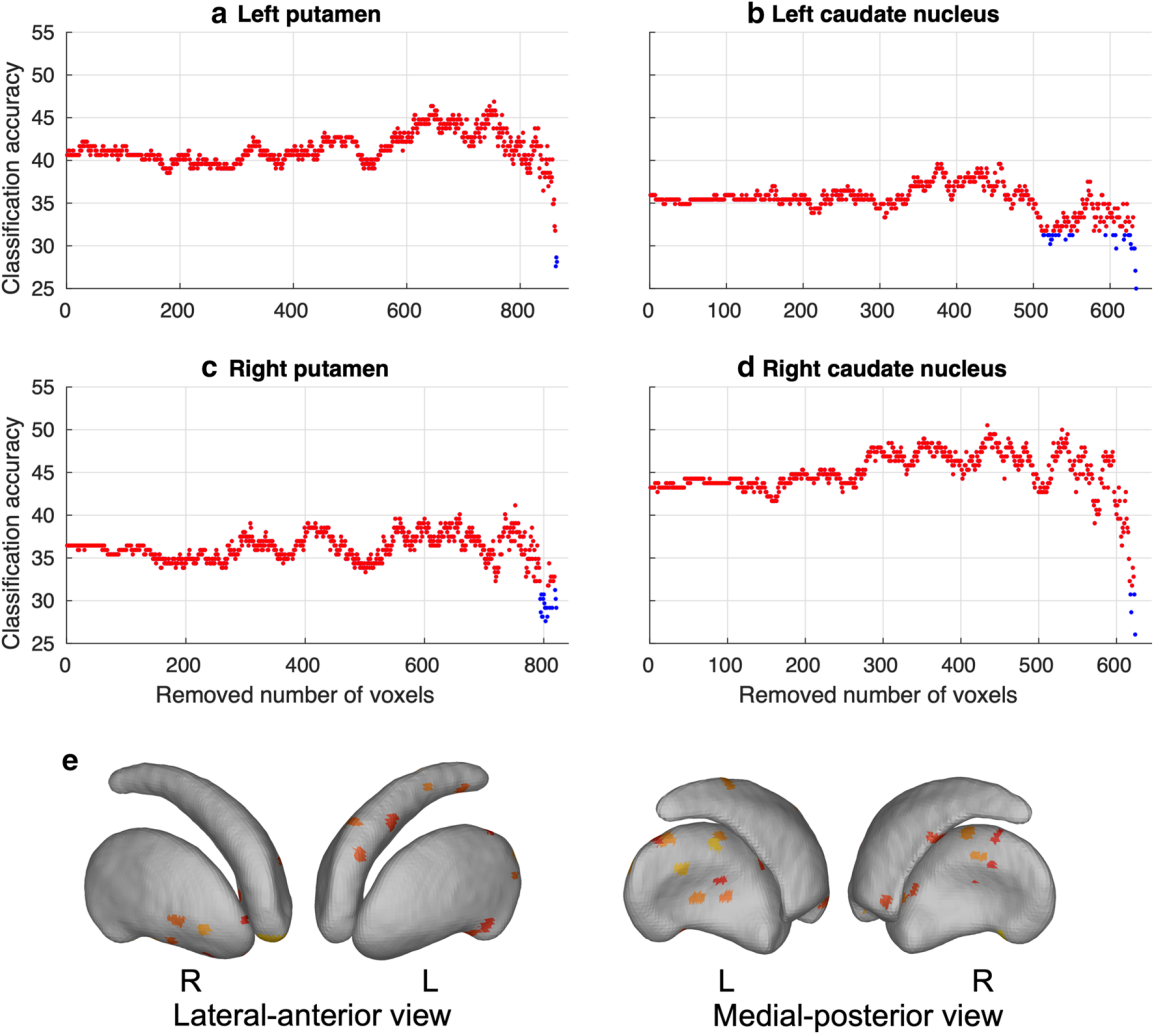
Sparsity analysis**.** Classification accuracy (in %) when iteratively removing voxels with the least sensitivity for **(a)** left putamen, **(b)** left caudate nucleus, **(c)** right putamen, and **(d)** right caudate nucleus. Red dots indicate FDR q < 0.05, blue dots not significant. Significant decoding can be found with low number of voxels in all striatal structures. **(e)** Show the 10 mostly selected voxel for each of the 4 nuclei during the backward selection method. N = 192 samples used for classification. L = left, R = right. Figures were made using SPM12 software (https://www.fil.ion.ucl.ac.uk/spm/software/spm12).

Maps of the classifier weights revealed that the voxels important for classification (i.e., the voxels with high positive or negative weight values) revealed a speckled pattern, consisting of discrete patches of small clusters distributed throughout the striatum (Fig. 2). In addition, the mean sensitivity map, calculated as the mean squared weight values across all sequence pairs, revealed that in each nucleus, the important voxels were scattered across the striatum with a limited amount of spatial clustering (Fig. 3b). Classification accuracy plateaued already quite early, considering only a relative low number of voxels for classification. For all striatal structures, significant classifications (q_FDR_ < 0.05) were maintained with as low as 4 and 5 voxels (Fig. 4). This finding was revealed by removing, recursively, voxels from the classification model with the least sensitivity in a cross-validated framework.

### Searchlight classification yielded similar results

**Figure 5:**
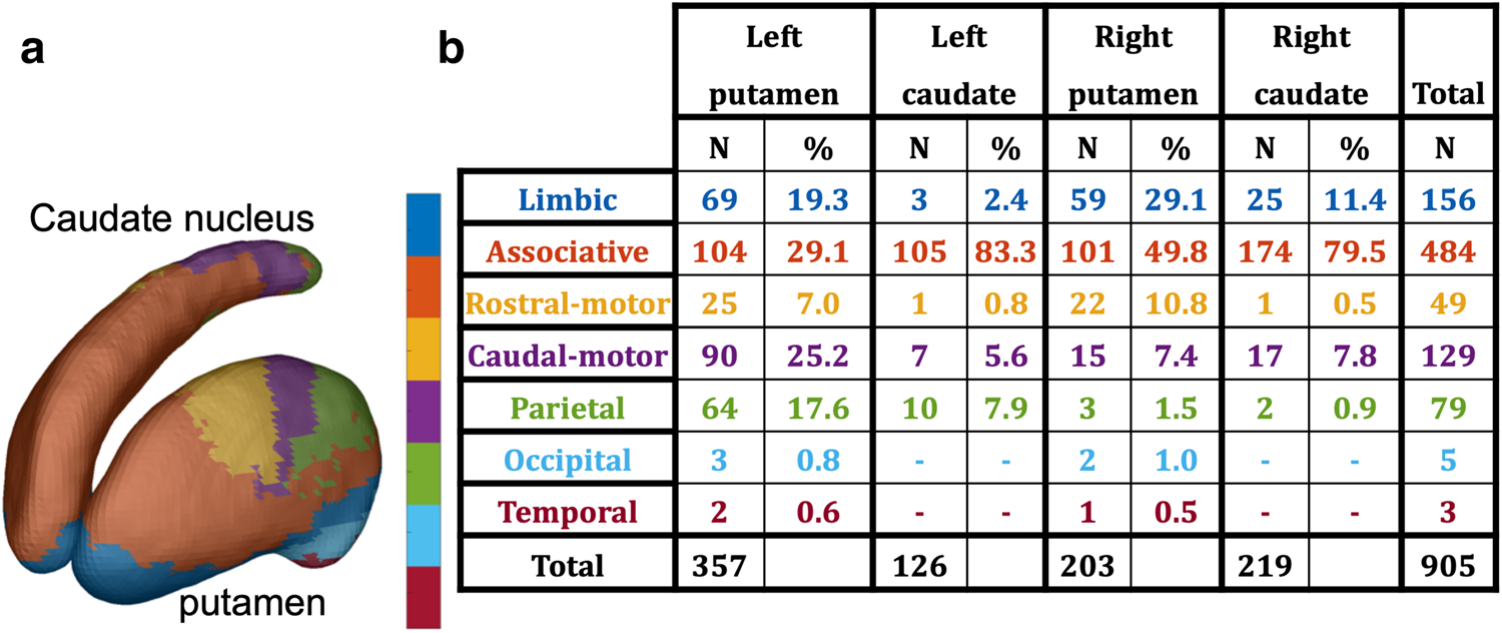
Distribution of significant searchlight. **(a)** outlines the 7 striatal sub-regions from the Oxford-GSK-Imanova Striatal Connectivity Atlas (Tziortzi et al. 2013). **(b)** lists the number (N) and percentage (%) of super-threshold voxels within each of the sub-regions. Figure were made using SPM12 software (https://www.fil.ion.ucl.ac.uk/spm/software/spm12).

In both, left and right striatum, significant between-sequence discrimination was also achieved within local spheres with 5 mm radius, so-called searchlights (Fig. 3c). The searchlight approach also revealed multiple patches where local patterns of striatal activity coded specific finger sequences. While the patches were dispersed across the striatum as the ROI-based classification, there was some degree of spatial clustering. To better characterize the underlying spatial pattern, we used the Oxford-GSK-Imanova Striatal Connectivity Atlas^26^ to quantify the distribution of significant searchlight voxels throughout the 7 sub-regions as outlined in the atlas (Fig. 5). Overall, the left putamen (n = 357) contained most voxels with significant discrimination property (q_FDR_ < 0.05, peak accuracy: 46.9%). Although classification showed evidence for coding discrete finger sequences in the rostral (n = 25) and caudal (n = 90) motor territory of left putamen (total n = 115, 32.2% of all significant voxels in this nucleus), the limbic, parietal, and associative territories of the left putamen each contained more than 60 voxels, which showed significant sequence classification in the searchlight procedure. In right putamen, the associative territory contained more significant classification voxels (n = 101, 49.8%) than the rostral (n = 22) and caudal (n = 15) motor territories (total n = 37, 18.2%). In the caudate nuclei, the majority of significant searchlight clusters were located in the associative territory of the respective nuclei (right caudate nucleus: n = 174; 79.5%; left caudate nucleus: n = 105; 83.3%) with additional contributions from limbic, motor and parietal territories.

### No sequence-specific differences in average striatal activation at single-voxel level

**Figure 6:**
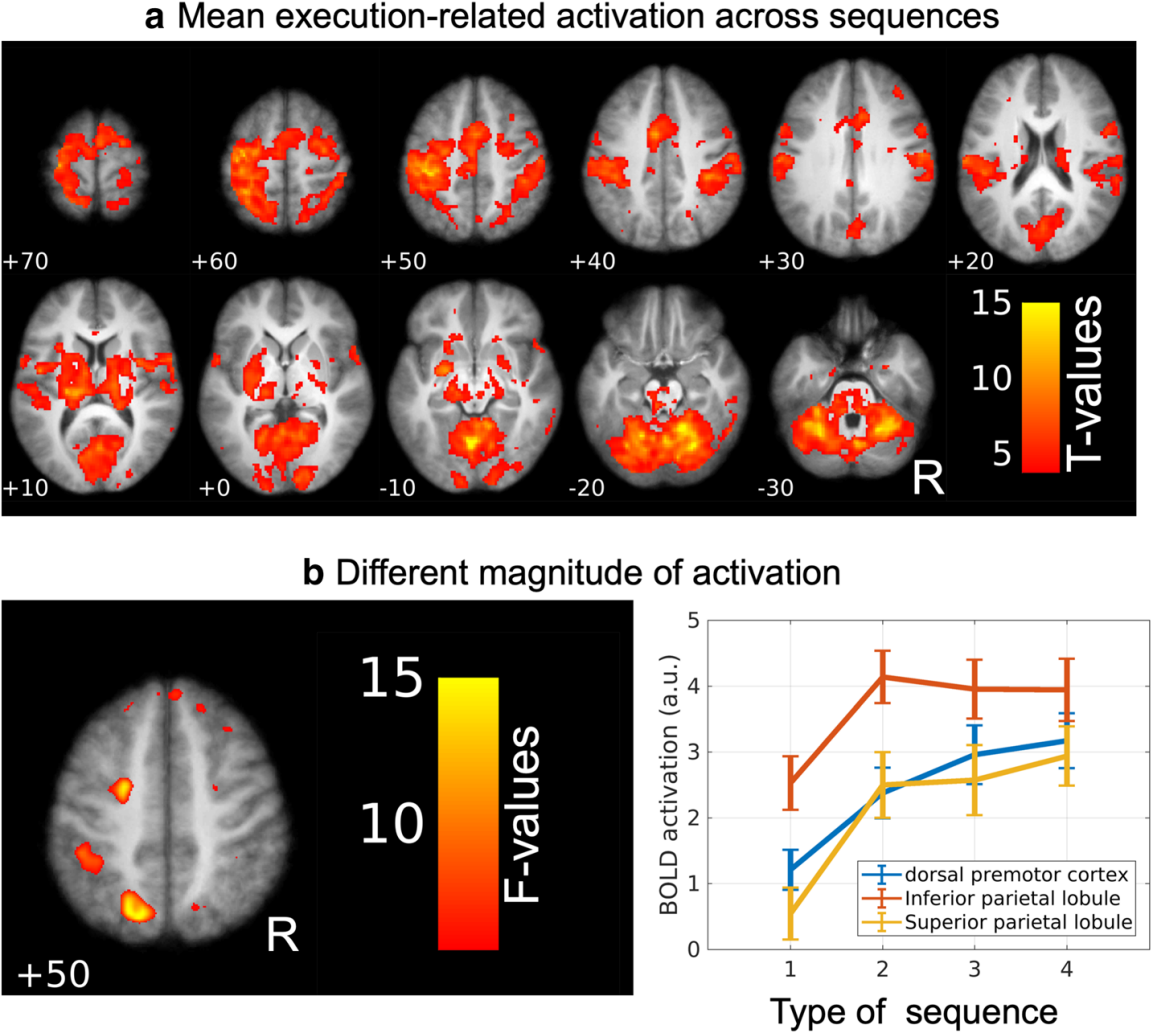
Average and differential activation. **(a)** Statistical parametric maps (SPM) showing the mean activation across the four different tapping-sequences (color-coded T-values). Numbers indicate the z-coordinate of the corresponding slice in MNI space. The map is thresholded at p < 0.001. **(b)** Cortical voxels showing a difference in mean task-related activity depending on the type of sequence (F-values). The map is thresholded at p < 0.001. R = right. N = 16 subjects. Figures were made using SPM12 software (https://www.fil.ion.ucl.ac.uk/spm/software/spm12).

Voxel-wise statistical parametric mapping showed task-related activation of a large cortico-basal ganglia-cerebellar motor network during the execution of finger sequences (Fig. 6a). In good agreement with previous work^1^, execution-related activation was found in primary and secondary motor regions, as well as a large extend of the striatum (Fig. 3a) and cerebellum. Three cortical clusters in the left hemisphere showed differential activation levels depending on the type of finger sequences (Fig. 6b). The clusters were located in the left superior and inferior parietal lobules and left dorsal premotor cortex. Post-hoc analysis showed that in all three clusters, the differential effect was caused by a higher activation during sequences 2–4 compared with sequence 1 (p ≤ 0.003).

No striatal voxels showed differential sequence effects (univariate “run” x “sequence” rm-ANOVA) even at the liberal threshold which is not corrected for multiple comparisons (uncorrected p < 0.001). Figure 3c shows a striatal surface representation of the mean striatal activity across sequences, showing activation primarily in the posterior motor related regions of putamen.

### Multivariate patterns also code for sequences during early learning

The multivariate pattern also showed unique sequence patterns during the first three runs 1–3, where the participants were still learning the task. This analysis showed that classification accuracies were significant in all structures: left putamen 41.7% correct (p < 0.001), left caudate nucleus 42.2% correct (p < 0.001), left putamen and caudate combined 46.4% correct (p < 0.001), right putamen 38.5% (p < 0.001) correct, right caudate 48.4% correct (p < 0.001), right putamen and caudate combined 52.1% (p < 0.001), all results survive correcting for multiple comparisons using the FDR method (q_FDR_ < 0.05). Interestingly, we note that accuracies are larger in the early learning compared with late learning phase, suggesting that sequences show a more distinct pattern in early learning as compared with stable performance has been reached. This observation warrants further study in future studies.

Using the fMRI data from runs 1–3, no bilateral voxels showed differential effects using univariate analysis (univariate “run” x “sequence” rm-ANOVA, uncorrected voxel level all p > 0.001). In addition, we tested for mean activation difference between runs 1–3 and runs 4–6 and found no voxels in the bilateral striatum with mean activation difference.

## Discussion

Extending the recent study by Pinsard et al.^25^, we found a widespread representation of sequential finger movements in human striatum. Multivariate pattern analysis of the execution-related distribution of BOLD signal changes revealed that discrete finger sequences were represented as multiple discrete patches of voxel clusters in left and right putamen and caudate nucleus in the absence of univariate differences in mean voxel-wise activity. Although the motor territory of left putamen contained multiple clusters that represent discrete finger sequences, this was the case for other striatal territories as well^27^. Especially the associative territories in the caudate nucleus and putamen, which receives corticostriatal inputs from prefrontal cortex, but also limbic and parietal striatal territories hosted representations of discrete finger movements. Our results significantly extend the paper by Wiestler & Diedrichsen^20^, which shows that sequential finger movements are coded by widely distributed activation patterns in frontoparietal premotor and primary sensorimotor areas.

Previous fMRI studies reported a shift in average sequence-related activity from associative (also called executive or prefrontal) striatal to motor territories in the putamen as motor performance becomes more proficient (reviewed by Doyon et al.^2^). In this study, our main focus was on the last three fMRI runs, during which task performance had reached a plateau in terms of movement time and accuracy. Therefore, one might have expected a clustering of sequence-specific multi-voxel activity patterns in the motor-related territories of the striatum. In our study, motor sequence representations were not confined to motor striatal territories. We rather found a speckled pattern of sequence representations with numerous clusters in the striatum displaying sequence-specific activity patterns. These clusters were bilaterally located mainly in motor and associative (prefrontal) territories, but also in parietal and limbic territories, indicating a widespread representation of discrete finger sequences in the human striatum. This implies that skilled sequential behaviour is widely represented in human striatum.

A few other studies have investigated the sequence specific patterns in the basal ganglia using approaches similar to what we applied in this study. Bednark et al.^24^ used MVPA to differentiate between sequences with a specific sequential rhythmic order (short or long index finger keypresses) with specific sequential order (keypresses with different fingers). Although they reported an extensive network of cortical motor regions with differential activity pattern, the activity pattern in the basal ganglia were not able to differentiate between order and rhythm^24^. A critical difference is that Bednark et al. focussed on the discrimination between order and rhythm, and not between different finger sequences as in the present study. Nambu et al.^21^ also used MVPA to discriminate between two different overlearned finger sequences of 20 keypresses using three fingers. They focussed on the preparatory phase but also report results from the execution phase of the task, but both phases did not show sequence specific patterns in the basal ganglia. Hence, both studies focused on different aspects of sequential motor control and thus cannot be compared directly to our study. Another noteworthy difference to our study is that we have utilized a between-subject MVPA design while Nambu et al.^21^ and Bednark et al.^24^ utilized within-subject MVPA analyses and ran group inference using parametric or non-parametric group analyses.

When interpreting this finding, one should bear in mind the nature of our task. Participants performed rather arbitrary sequences of button presses with their fingers. Unlike piano playing or typing, the presses did not serve a goal (i.e. to produce a melody or a string of letters) and as such were not clearly goal-related. The sequence learned in the study can also be conceptualized as habitual behavioural routines. In addition to learning motor skills, the striatum is also crucial for habit learning. Studies in rodents have consistently shown a striatal involvement during habit formation^28,29^. The dual nature of our task, sharing a motor skill and a habit component, may at least partially account for the widespread striatal presentation. Interestingly, we found high involvement of all striatal structures during the early learning of the task (runs 1–3), where the classification accuracies were higher as compared with the later runs (runs 4–6). We attribute the better classification accuracy during the first three runs to a higher cognitive load. Since participants were not trained on the sequences prior to scanning, they were relatively naïve and task-related activity during the first three runs belong to the very early phase of motor sequence learning, when participants still had to maintain the visually presented sequence in short-term memory prior to motor execution^25^. Hence, task performance during the first fMRI runs most likely engaged a mixture of cognitive processes, including working memory, visuospatial processing, and sensorimotor representations^25^. The cortical regions involved in these processes send projections to different (but overlapping) territories of the striatum. We argue that more cortical networks were engaged in the early learning phase (runs 1–3), involving a larger extent of the striatum, resulting in a more unique representational pattern and thus, higher classification accuracies. This interpretation must be verified in future studies, for instance by asking participants to memorize, visualize and perform overlearned motor sequences. This should also increase classification accuracy compared to simple motor performance by increasing cognitive load and reverting automated overlearned task performance back to a non-automatized controlled mode of task performance.

The temporal dynamics of striatal activity patterns during motor skill and habit learning may provide an additional clue. Motor skills show an initial dependence on visuospatial allocentric representations that gradually shift towards a reliance on sensorimotor egocentric representations^2^. Analogous dynamics have been reported in rodent studies on habit formation with the dorsomedial striatum being crucial for initial acquisition and dorsolateral striatum for consolidation^28,29^ and a shift to dorsal striatal control over behaviour has been identified as an important mechanism underlying addiction (reviewed by Everitt and Robbins^30^).

Since finger sequences had only been trained during the first three fMRI runs, it is possible that sequence execution still relied on both, visuospatial and sensorimotor sequence representations in the striatum during the late fMRI runs (i.e., runs 4–6) despite of stable task performance. Representations may become more confined to sensorimotor representations after more prolonged motor sequence training. These alternatives need to be systematically studied in future fMRI studies involving motor sequence training over days or weeks.

The striatum contains discrete and anatomically segregated territories which receive cortico-striatal input from distinct cortical regions and show a substantial degree of synaptic convergence^27,31^. The convergence of anatomically segregated cortical projections in the striatum enables the parallel funnelling of information from different cortical areas, and provides a critical important anatomical foundation for the basal ganglia´s ability to reinforce appropriate and suppress inappropriate behaviours in sensorimotor, associative and affective tasks^32^. We found that many striatal patches located in multiple territories of the right and left striatum represent sequential finger movements. This begs the question how sequence-related information represented in multiple parallel cortico-striatal loops is integrated to facilitate the fast, fluent and correct generation of a specific action sequence. Integration may occur through cortico-cortical connections, given the fact that multiple frontoparietal areas contain sequence representations^20^ and these areas are densely connected by reciprocal cortico-cortical loops^33^. It has been proposed that the basal ganglia serve as a “general purpose trainer for cortico-cortical connections”^34^.

Complementing cortico-cortical mechanisms, several lines of research have pinpointed several subcortical mechanisms through which information can be effectively integrated among cortico-basal ganglia-thalamo-cortical loops. The (monosynaptic) cortico-striatal input and (polysynaptic) striato-cortical output projections show a substantial amount of divergence, forming split-circuits which enable open-loop functional integration across striatal territories^35^. Neighbouring cortical areas show overlap regarding corticostriatal inputs, but there are also patches in the striatum (i.e., convergence zones) which receive common input from distant cortical sites^36,37^. Tracer studies in the monkey showed overlapping and interweaved cortico-striatal projections from fronto-parietal regions, which are mutually connected through cortico-cortical projections^38–40^. Divergence has also been demonstrated for striato-cortical projections. In cebus monkeys, a patch in the ventral of the putamen projects to the motor cortex through pallido-thalamo-cortical projections, but do not receive corticostriatal input from the motor cortex, providing a limbic route from the amygdala to motor cortex^41^. In addition to cortico-striato-cortical mechanisms, a substantial deal of integration across striatal territories employs subcortico-subcortical loops involving the thalamus and brainstem nuclei such as the midbrain dopaminergic nuclei^42–44^. A recent optogenetic stimulation study in rodents provided evidence for a unidirectional limbic-to-motor pathway through which the ventral striatum influences the motor cortex and hereby the motor cortico-basal ganglia loop^45^. Together, the multiple between-circuit interactions within the basal ganglia provide a neuroanatomical basis for a synergistic integration of multiple sequence representations in the human basal ganglia circuities.

Our results suggest that the representation of motor sequences is widely distributed in the human basal ganglia. This observation is of relevance with respect to the pathophysiology of Parkinson’s disease (PD)^46^. PD is a neurodegenerative disease which results in a progressive neurodegeneration of the nigrostriatal dopaminergic input to the basal ganglia with a caudal-to-rostral gradient in terms of the severity of neurodegeneration. Dopaminergic denervation of the striatum affects the execution of sequential finger movements with patients displaying marked hesitations between the movement segments compared with healthy controls^47^. Task-related fMRI has been used in several studies to map changes in regional brain activity, when patients produced overlearned motor sequences^48,49^. When motor sequences were self-initiated, the sensorimotor posterior putamen showed a reduced movement related activity during motor execution in unmedicated de novo patients with PD^48^. In contrast, task related activation of the associative anterior putamen, a region that has been linked to sequence planning in healthy individuals^50^, was normal during the planning phase of the motor sequence task, along with an overactivity of the dorsolateral prefrontal cortex task^48^. Together these findings are compatible with the notion of multiple sequence representations in the basal ganglia. A motor representation in the posterior putamen is affected, but a cognitive representation in the anterior putamen is relatively spared and can contribute to compensate for the impaired motor representation in PD^48^.

In conclusion, our multivariate neuroimaging approach provides an integrated rather than a segregated account on how discrete finger sequences are represented in the human striatum. We show that distributed task-related activity patterns in multiple bilateral striatal patches represent sequence-specific information. This finding significantly extends previous fMRI studies using univariate analyses of average voxel-wise activity, suggesting a more confined engagement of the posterior and middle putamen. We infer from our findings that multiple cortico-basal ganglia-thalamo-cortical loops jointly contribute to motor sequence control code, supporting the role of the basal ganglia in integrating motor, associative and limbic aspects in the control of sequential behaviour.

## Methods

### Volunteers

16 healthy volunteers (8 females, mean age 22.3 ± 3.0) with no history of neurological and psychiatric disorders participated in this study. The study was approved by the Copenhagen Ethics Committee (H-1-2013-007) and carried out in accordance with the Declaration of Helsinki. All subjects gave written informed consent before participating in the study. Right-handedness was confirmed with the Edinburgh Handedness inventory^51^.

### Discrete finger sequence task

Each participant performed a set of four discrete finger sequences each of which was composed of five button presses (Fig. 1a). Participants were instructed to produce the sequence as fast and accurate as possible. The sequences varied by the number of direction changes and jumps included. Level 1 was a simple ascending or descending sequence without jumps or direction changes. Level 2 included one directional change and two jumps. Level 3 comprised two direction changes and three jumps. Level 4 consisted of three directional inversions and four jumps. Two sets of sequences, mirrored versions of each other, were constructed. Each participant performed one of the two sets of sequences (randomly assigned to the subjects). Participants did not train the sequences before scanning to be able to capture learning-induced performance improvements during the fMRI experiment.

The timeline of a single trial is illustrated in Fig. 1a. In one trial, participants performed the same sequence three times. At the beginning of each trial, the sequence was visually indicated to the participant by presenting a schematic drawing of the dorsal surface of the right hand with a dot jumping from fingertip to fingertip. Participants were instructed to memorize the five-movement sequence. The hand was replaced after 3 s by a white central fixation cross. Motor execution was visually cued at a variable interval, ranging from 1 to 6 s, by presenting a circle around the fixation cross. Participants were instructed to produce the indicated sequence as fast and accurate as possible upon appearance of the circle. The circle was shown for 3 s, Thereafter the fixation cross was presented again without the circle, instructing participants to rest until the next circle appeared. After the third iteration, the screen turned black for 1 s hereafter visual feedback was given to the subject, presented as three smileys. The left-to-right order of the smileys corresponded to the temporal order of the sequences performed in the preceding trial. A happy green smiley indicated correct and a sad red smiley incorrect performance. Again, a black screen was presented for a variable interval of 1–6 s to separate two consecutive trials. Median trial duration (including inter-trial interval) was 29 s (23–36 s).

### Magnetic resonance imaging

Whole-brain functional brain activity was scanned using a 3 T Philips Achieva scanner and a 32-channel head coil (Philips, Best, The Netherlands) and an echo planar imaging (EPI) sequence (TR/TE = 2,200/30 ms, flip-angle = 80°, 42 axial slices) with a 2.64 × 2.64 × 3 mm voxel resolution. Each participant underwent six fMRI runs (each 218 volumes lasting 8 min). During a single fMRI run, each of the four finger sequences was repeated four times in pseudorandom order (16 trial blocks in total). The button presses were captured with a custom made 5-button device. The task was presented using PsychoPy (https://www.psychopy.org/) version 1.75.01.

A high-resolution three-dimensional T1-weighted image (TR/TE = 5,980/2.71 ms, flip-angle = 8°) was obtained of the whole brain at 0.85 mm isotropic resolution (matrix size 256 × 256 × 190). The T1-weighted images were used for brain tissue segmentation and for creating a group-specific mean T1-image. During the MRI session, head motion was minimized with head foam cushions and the cardiac cycle was measured with an infrared pulse oximeter attached to the left index finger. Respiration was measured with a pneumatic thoracic belt.

### Data processing

#### Behavioural data

In each participant, we calculated mean movement accuracy and total movement time for each type of sequence and fMRI run. A finger sequence was performed correctly, if it was performed in the correct order and total movement time was shorter than 2.5 s. Movement time was defined as the time that elapsed between the first and last button press in a sequence and was only calculated for the correct trials.

#### Structural MRI data

The individual high resolution T1-weighted images were used to segment out the putamen and caudate nucleus using FSL’s FIRST (https://fsl.fmrib.ox.ac.uk/fsl/fslwiki/FIRST). Following segmentation, the whole brain structural image was normalized to MNI space using SPM12 and the putamen and caudate nucleus masks were warped using the same transformation parameters. In standard space, average masks across subjects of left and right putamen and caudate nucleus were created. Each mask was thresholded, including all voxels where at least 50% of the subjects showed overlap. Finally, masks were resliced to 2 mm isotropic voxel sizes to match the final resolution of the functional data. In addition, we constructed masks for left and right striatum, respectively, by combining putamen and caudate nucleus in each hemisphere separately.

#### Functional MRI data

Functional MRI data were pre-processed using SPM12 software (https://www.fil.ion.ucl.ac.uk/spm/software/spm12). The functional images were slice-time corrected, re-aligned, de-spiked (in-house software), normalized to MNI space, and smoothed with a 5 mm FWHM isotropic kernel. The data was analysed with a general linear model (GLM) where regressors were generated by convolving the onset times for the visual task instruction (hand with jumping dots), finger sequence execution (circle presentation), and feedback (three smileys) with the canonical HRF and its temporal derivative. Separate regressors were created for correct and incorrect sequence executions. In addition, the realignment parameters, their temporal lagged version, and all squared (24 in total per run) were added as nuisance regressors. We added 18 regressors per run reflecting the Fourier expansion of the cardiac and respiratory phases and their interaction, as well as the heart rate variability and respiratory volume per time^52–54^. To reduce residual head motion artefacts, volumes with more than 1 mm framewise displacement^55^ were nulled with volume removing regressors. To analyse the main effect of sequence execution, we set up a contrast to average the beta estimates for the four sequences. The beta estimates were only averaged for the last 3 runs where stable performance was obtained.

### Sequence classification

At the subject level, voxel-wise GLM parameter estimates (beta values) for the correct execution of each sequence and run were extracted for each of the basal ganglia masks and used for multivariate classification. We asked whether the sequences could be decoded from activity patterns in each of the basal ganglia sub-structures during the period where subjects had reached stable behavioural performance. Analysis of the behavioural performance showed that in the last 3 experimental runs, there were no difference in movement time and accuracy, so the last 3 runs were considered for classification. This resulted in 192 multivariate samples (16 subjects × 3 runs × 4 sequences) per mask. Support Vector Machine (SVM) classifiers were trained to discriminate between the different sequences using LIBSVM (https://www.csie.ntu.edu.tw/~cjlin/libsvm/). This was done in a leave-one-sample-out cross-validation scheme, where each sample in turn was left out during model training and used for testing. We used the linear kernel with c = 1 (default setting). The mean accuracy was then calculated across the full number of samples. LIBSVM allows for multi-class classification using a 1-against-1 strategy where classifiers are constructed for each pair of classes (for a 4 class problem this results in 6 different classifiers). When performing the classification for test data, the data were evaluated with each classifier and assigned the class with the highest vote among classifiers. Prior to training, data from each subject and run were de-meaned by subtracting the voxelwise mean across the four sequences for that run. The training procedure used all voxels in each of the 4 considered basal ganglia masks separately. In addition, we tested whether classification improved when combining putamen and caudate nucleus in each hemisphere. For visualization of the spatial distribution of the classifier weights, we trained a model using all samples and visualized the resulting weights on the surface of each of the structures and for each of the pair of sequences (Fig. 2 and 3b).

### Backward model selection

To reveal the dimensionality of the underlying discriminative patterns, we performed a voxel selection procedure within each of the striatal structures. Again, this was done using a leave-one-sample-out procedure, where for each sample left out the following procedure was performed. The classifiers were trained with the remaining voxels and the mean sensitivity, defined as the mean square of the classifier weights across classifiers (different sequence pairs), of each voxel were calculated. The voxel with the least sensitivity was eliminated from the set of voxels, and the procedure was repeated until only a single voxel was left in the model. This procedure was repeated for each sample used as test sample. The mean classifier accuracy was calculated for each number of left-out voxels. A classification was deemed significant for a given number of voxels in the model at the FDR q < 0.05 level, see Fig. 4.

### Searchlight

Within each of the structures, we performed a searchlight classification. In this procedure, each voxel within the striatal structures was used as the centre of a sphere and voxels within a radius of 5 mm were used for classification and the resulting classification accuracy was then assigned to the centre of the sphere.

### Statistical analysis

#### Behavioural data

Mean accuracy and movement time were analysed in separate repeated measures analysis of variance (rm-ANOVA) with the factors *fMRI run* and *type of sequence.* The Greenhouse–Geisser correction method was applied, if necessary, to correct for non-sphericity. Dependent on significant main effects or interaction terms in the ANOVA, we performed post-hoc T-tests. Significance level was set at p < 0.05. The behavioural analyses were performed with SPSS (IBM Corp. Released 2013. IBM SPSS Statistics for Macintosh, Version 22.0. Armonk, NY: IBM Corp).

#### Univariate statistical parametric mapping

At the group level, we performed a 1-sample t-test to find the main effect of sequence execution. In addition, we performed a “run” x “sequence” repeated-measures ANOVA to analyse the differential effect of sequence at the whole brain level. These analyses only used the beta estimated for the last 3 runs where stable performance was observed. We used a cluster-forming threshold of p < 0.001 and considered clusters significant when p < 0.05 controlling for multiple comparisons using family-wise error correction.

#### SVM classification

The significance levels were evaluated with permutation tests where 10.000 leave-one-sample-out procedures were performed as outlined above, but where training labels were randomly permuted and accuracies for the 10.000 random permutations were used for building a NULL-distribution. To account for multiple comparisons across the 6 regions of interests (left and right putamen, caudate nucleus, and putamen + caudate nucleus), the classifications were considered significant if they survived false-discovery-rate q_FDR_ < 0.05.

#### Searchlight analyses

To assess the significance level, we repeated the analyses 10 times per voxel for the bilateral putamen and caudate, but with permuted sequence classes, resulting in 29,490 random tests across voxels, which were all used for the null-distribution. The searchlight classifications were considered significant at a q_FDR_ < 0.05 level, which corresponded to an accuracy of 33.34% correct and uncorrected p-value = 0.015. We used the Oxford-GSK-Imanova Striatal Connectivity Atlas^26^ for quantitatively describing how the significant searchlight voxels were distributed within the structures.

## Data Availability

The data supporting the findings of this work are available from the corresponding author upon reasonable request.

## References

[1] Hardwick RM, Rottschy C, Miall RC, Eickhoff SB (2013). A quantitative meta-analysis and review of motor learning in the human brain. Neuroimage.

[2] Doyon J, Gabitov E, Vahdat S, Lungu O, Boutin A (2018). Current issues related to motor sequence learning in humans. Curr. Opin. Behav. Sci..

[3] Lehéricy S (2005). Distinct basal ganglia territories are engaged in early and advanced motor sequence learning. Proc. Natl. Acad. Sci. USA.

[4] Steele CJ, Penhune VB (2010). Specific Increases within Global Decreases: A Functional Magnetic Resonance Imaging Investigation of Five Days of Motor Sequence Learning. J. Neurosci..

[5] Coynel D, Marrelec G, Perlbarg V, Pélégrini-issac M (2010). NeuroImage dynamics of motor-related functional integration during motor sequence learning. Neuroimage.

[6] Floyer-Lea A, Matthews PM (2005). Distinguishable brain activation networks for short- and long-term motor skill learning. J. Neurophysiol..

[7] Albouy G, King BR, Maquet P, Doyon J (2013). Hippocampus and striatum: Dynamics and interaction during acquisition and sleep-related motor sequence memory consolidation. Hippocampus.

[8] Debas K (2014). Off-line consolidation of motor sequence learning results in greater integration within a cortico-striatal functional network. Neuroimage.

[9] Wymbs NF, Bassett DS, Mucha PJ, Porter MA, Grafton ST (2012). Differential recruitment of the sensorimotor putamen and frontoparietal cortex during motor chunking in humans. Neuron.

[10] Miyachi S, Hikosaka O, Lu X (2002). Differential activation of monkey striatal neurons in the early and late stages of procedural learning. Exp. Brain Res..

[11] Hikosaka O (1999). Parallel neural networks for learning sequential procedures. Trends Neurosci..

[12] Miyachi S, Hikosaka O, Miyashita K, Kárádi Z, Rand MK (1997). Differential roles of monkey striatum in learning of sequential hand movement. Exp. Brain Res..

[13] Yin HH (2009). Dynamic reorganization of striatal circuits during the acquisition and consolidation of a skill. Nat. Neurosci..

[14] Jin X, Tecuapetla F, Costa RM (2014). Basal ganglia subcircuits distinctively encode the parsing and concatenation of action sequences. Nat. Neurosci..

[15] Martiros N, Burgess AA, Graybiel AM (2018). Inversely active striatal projection neurons and interneurons selectively delimit useful behavioral sequences. Curr. Biol..

[16] Santos FJ, Oliveira RF, Jin X, Costa RM (2015). Corticostriatal dynamics encode the refinement of specific behavioral variability during skill learning. Elife.

[17] Haxby JV, Connolly AC, Guntupalli JS (2014). Decoding neural representational spaces using multivariate pattern analysis. Annu. Rev. Neurosci..

[18] Diedrichsen J, Kriegeskorte N (2017). Representational models: a common framework for understanding encoding, pattern-component, and representational-similarity analysis. PLoS Comput. Biol..

[19] Diedrichsen J, Yokoi A, Arbuckle SA (2017). Pattern component modeling: a flexible approach for understanding the representational structure of brain activity patterns. Neuroimage.

[20] Wiestler T, Diedrichsen J (2013). Skill learning strengthens cortical representations of motor sequences. Elife.

[21] Nambu I (2015). Decoding sequential finger movements from preparatory activity in higher-order motor regions: a functional magnetic resonance imaging multi-voxel pattern analysis. Eur. J. Neurosci..

[22] Yokoi A, Arbuckle SA, Diedrichsen J (2018). The role of human primary motor cortex in the production of skilled finger sequences. J. Neurosci..

[23] Kornysheva K, Diedrichsen J (2014). Human premotor areas parse sequences into their spatial and temporal features. Elife.

[24] Bednark JG, Campbell MEJ, Cunnington R (2015). Basal ganglia and cortical networks for sequential ordering and rhythm of complex movements. Front. Hum. Neurosci..

[25] Pinsard B (2019). Consolidation alters motor sequence- specific distributed representations. Elife.

[26] Tziortzi AC (2014). Connectivity-based functional analysis of dopamine release in the striatum using diffusion-weighted MRI and positron emission tomography. Cereb. Cortex.

[27] Alexander GE, DeLong MR, Strick PL (1986). Parallel organization of functionally segregated circuits linking basal ganglia and cortex. Annu. Rev. Neurosci..

[28] Thorn CA, Atallah H, Howe M, Graybiel AM (2010). Differential dynamics of activity changes in dorsolateral and dorsomedial striatal loops during learning. Neuron.

[29] Smith KS, Graybiel AM (2013). A dual operator view of habitual behavior reflecting cortical and striatal dynamics. Neuron.

[30] Everitt BJ, Robbins TW (2013). From the ventral to the dorsal striatum: Devolving views of their roles in drug addiction. Neurosci. Biobehav. Rev..

[31] Delong MR (1984). Functional organization of the basal ganglia: contributions of single-cell recording studies. Ciba Found. Symp..

[32] Bar-Gad I, Morris G, Bergman H (2003). Information processing, dimensionality reduction and reinforcement learning in the basal ganglia. Prog. Neurobiol..

[33] Rizzolatti G, Luppino G, Matelli M (1998). The organization of the cortical motor system: new concepts. Electroencephalogr. Clin. Neurophysiol..

[34] Hélie S, Ell SW, Ashby FG (2015). Learning robust cortico-cortical associations with the basal ganglia: an integrative review. Cortex.

[35] Joel D, Weiner I (1994). The organization of the basal ganglia-thalamocortical circuits: open interconnected rather than closed segregated. Neuroscience.

[36] Averbeck BB, Lehman J, Jacobson M, Haber SN (2014). Estimates of projection overlap and zones of convergence within frontal-striatal circuits. J. Neurosci..

[37] Jarbo K, Verstynen TD (2015). Converging structural and functional connectivity of orbitofrontal, dorsolateral prefrontal, and posterior parietal cortex in the human striatum. J. Neurosci..

[38] Gerbella M, Borra E, Mangiaracina C, Rozzi S, Luppino G (2015). Corticostriate projections from areas of the ‘lateral grasping network’: evidence for multiple hand-related input channels. Cereb. Cortex.

[39] Selemon LD, Goldman-Rakic PS (1988). Common cortical and subcortical targets of the dorsolateral prefrontal and posterior parietal cortices in the rhesus monkey: evidence for a distributed neural network subserving spatially guided behavior. J. Neurosci..

[40] Yeterian EH, Van Hoesen GW (1978). Cortico-striate projections in the rhesus monkey: The organization of certain cortico-caudate connections. Brain Res..

[41] Kelly RM, Strick PL (2004). Macro-architecture of basal ganglia loops with the cerebral cortex: use of rabies virus to reveal multisynaptic circuits. Prog. Brain Res..

[42] McHaffie JG, Stanford TR, Stein BE, Coizet V, Redgrave P (2005). Subcortical loops through the basal ganglia. Trends Neurosci..

[43] Haber SN, Calzavara R (2009). The cortico-basal ganglia integrative network: the role of the thalamus. Brain Res. Bull..

[44] Draganski B (2008). Evidence for segregated and integrative connectivity patterns in the human Basal Ganglia. J. Neurosci..

[45] Aoki S (2019). An open cortico-basal ganglia loop allows limbic control over motor output via the nigrothalamic pathway. Elife.

[46] Doyon J (2008). Motor sequence learning and movement disorders. Curr. Opin. Neurol..

[47] Weiss P, Stelmach GE, Hefter H (1997). Programming of a movement sequence in Parkinson’s disease. Brain.

[48] Martin JA (2019). Disentangling motor planning and motor execution in unmedicated de novo Parkinson’s disease patients: An fMRI study. NeuroImage Clin..

[49] Herz DM, Eickhoff SB, Løkkegaard A, Siebner HR (2014). Functional neuroimaging of motor control in parkinson’s disease: A meta-analysis. Hum. Brain Mapp..

[50] Jankowski J, Scheef L, Hüppe C, Boecker H (2009). Distinct striatal regions for planning and executing novel and automated movement sequences. Neuroimage.

[51] Oldfield RC (1971). The assessment and analysis of handedness: The Edinburgh inventory. Neuropsychologia.

[52] Glover GH, Li TQ, Ress D (2000). Image-based method for retrospective correction of physiological motion effects in fMRI: RETROICOR. Magn. Reson. Med..

[53] Birn RM, Smith MA, Jones TB, Bandettini PA (2008). The respiration response function: The temporal dynamics of fMRI signal fluctuations related to changes in respiration. Neuroimage.

[54] Chang C, Cunningham JP, Glover GH (2009). Influence of heart rate on the BOLD signal: The cardiac response function. Neuroimage.

[55] Power JD, Barnes KA, Snyder AZ, Schlaggar BL, Petersen SE (2012). Spurious but systematic correlations in functional connectivity MRI networks arise from subject motion. Neuroimage.

